# Data on modeling of UV/Na_2_S_2_O_8_/FeS_2_ process in amoxicillin removal using Box-Behnken methodology

**DOI:** 10.1016/j.dib.2018.06.109

**Published:** 2018-07-02

**Authors:** Roshanak Rezaei Kalantary, Massuomeh Rahmatinia, Masoud Moradi

**Affiliations:** a Department of Environmental Health Engineering, School of Public Health, Iran University of Medical Sciences, Tehran, Iran; b Research Center for Environmental Health Technology, Iran University of Medical Sciences, Tehran, Iran; c Research center for Environmental Determinants of Health, Kermanshah University of Medical Sciences, Kermanshah, Iran

**Keywords:** Amoxicillin, Box-Behnken, UV/Na_2_S_2_O_8_/FeS_2_

## Abstract

Among the pharmaceutical compounds, antibiotics have been paid specific consideration, due to their acute and chronic toxic effects on organisms. Amoxicillin (AMX) is used widely for treatment of bacterial infections. About 80% of amoxicillin excreted unchanged and enters the aquatic environment through different routes including disposal of municipal wastewaters, hospital wastewaters and farm wastewaters. In this study degradation of amoxicillin by UV/Na_2_S_2_O_8_/FeS_2_ process was evaluated. According to the results, the R-squared and adjusted R-squared were 0.9877 and 0.9828, respectively. The AMX removal efficiency was 93% at optimum conditions. Thus, UV/Na_2_S_2_O_8_/FeS_2_ process is a useful process for amoxicillin removal.

**Specifications Table**

**Table:** 

Subject area	Environmental engineering
More specific subject area	Advanced oxidation process
Type of data	Figures and tables
How data was acquired	All degradation tests were done in a reactor batch (Volume of 1 L), equipped with a UV-C lamp (16 W). Three level of each parameter was evaluated using BOX-Behnken design.
	A High Liquid Performance Chromatography (HPLC) was used for the determination of AMX concentration.
Data format	Analyzed
Experimental factors	Measuring of AMX concentrations under various levels of initial AMX concentration, solution pH, Persulfate concentration, dose of FeS_2_ and contact time to obtain optimum AMX removal from aqueous solutions.
Experimental features	Optimization of AMX degradation using BOX-Behnken design.
Data source location	Iran University of Medical sciences, Tehran, Iran
Data accessibility	Data are available within paper.

**Value of data**

• The synthesized catalyst has properties include earth abundant, low cost, high absorption coefficient and good photocatalytic activity. Also, pyrite catalyst is reusable.
• This research shows a statistical method (Box-Behnken design) to optimize AMX removal from aqueous solution.
• The obtained data will be appropriate for AMX removal from water and wastewater.

## 1 Data

**Fig. 1:**
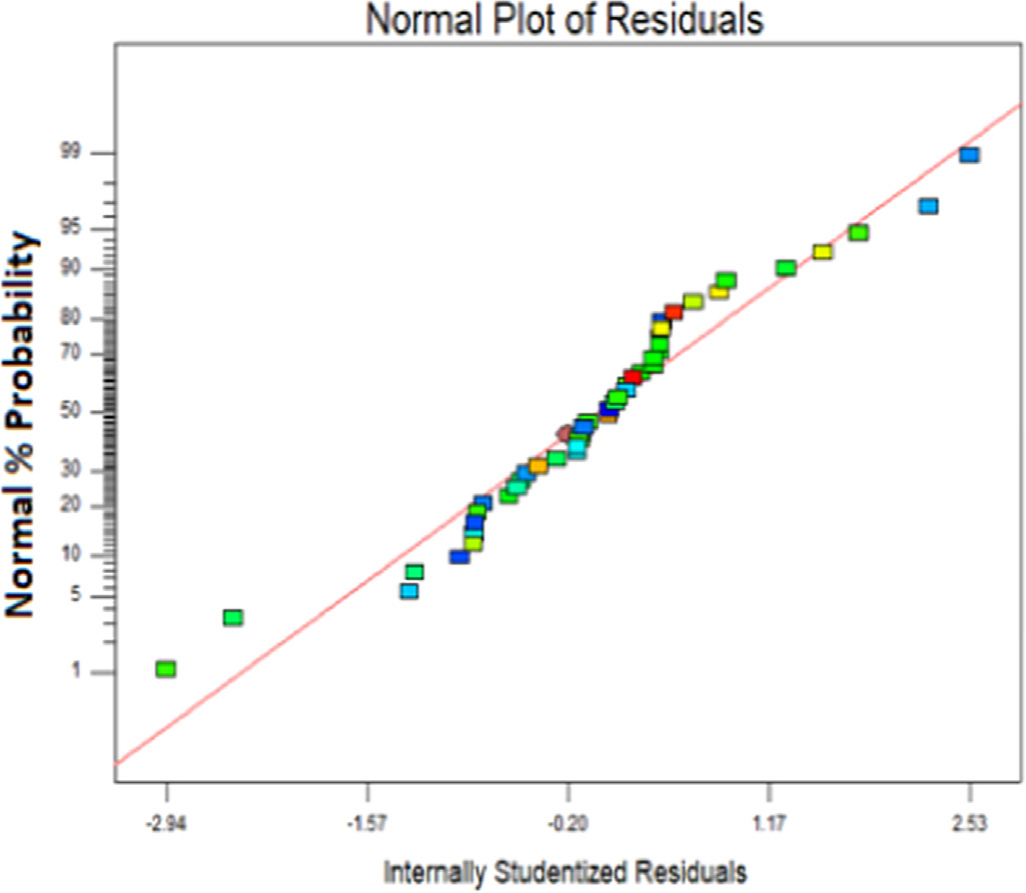
Normal probability plot of studentized residuals.

**Fig. 2:**
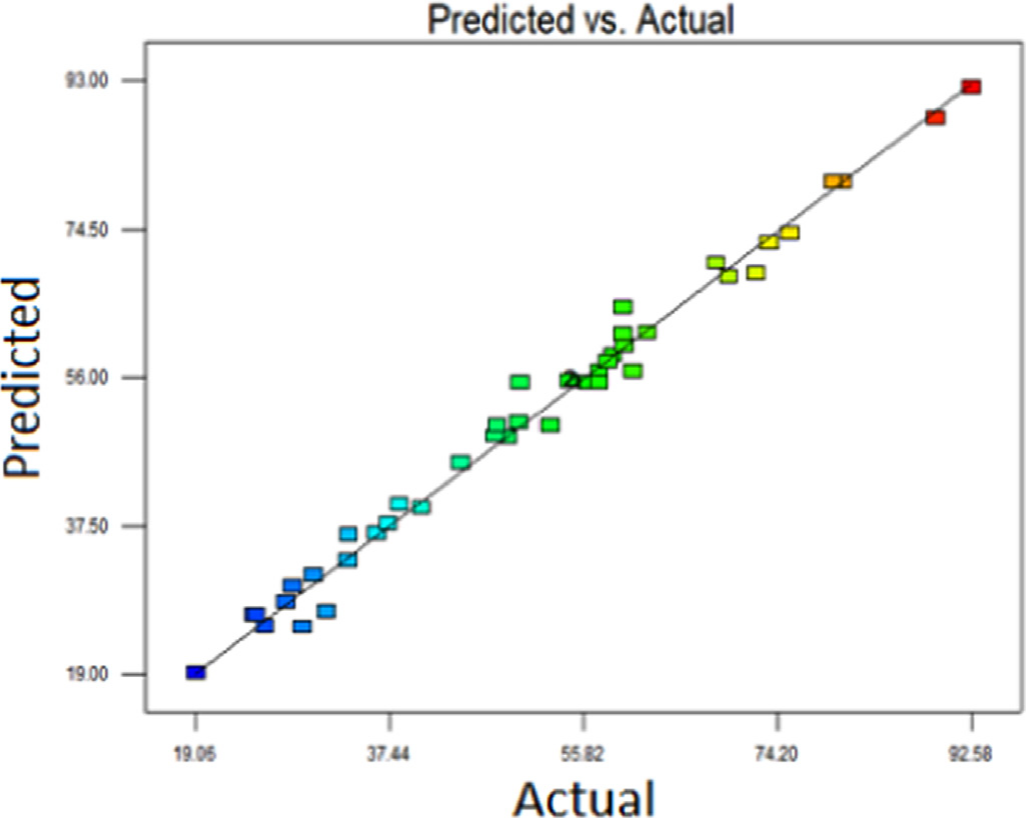
Actual and predicted data of AMX removal.

**Fig. 3:**
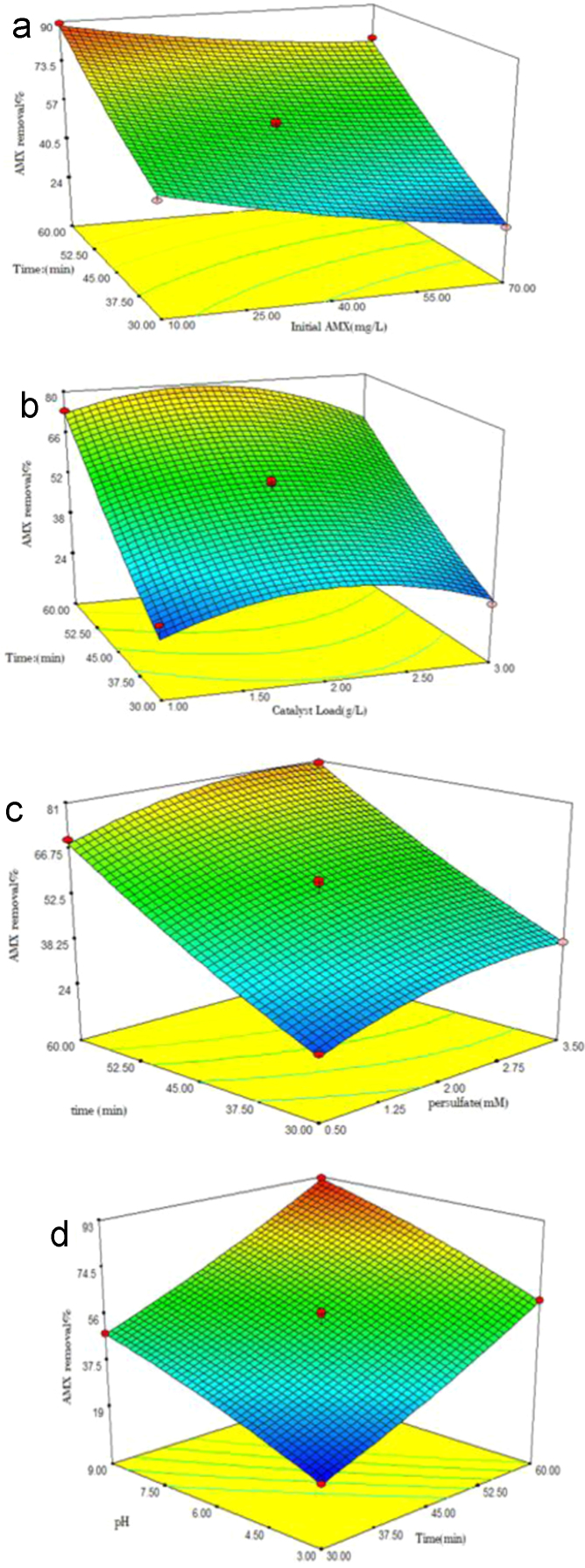
Response surface plots for AMX removal by UV/Na_2_S_2_O_8_/FeS_2_ (a) AMX removal versus initial AMX and time (b) AMX removal versus AMX catalyst load and time (c) AMX removal versus persulfate dose and time (d) AMX removal versus pH and time.

The level of variables and their codes are shown in Table 1. For optimization of UV/Na_2_S_2_O_8_/FeS_2_ process, Box-Behnken design (BBD) was applied as a response surface method [1], [2], [3]. The adequacy of the model was checked using analysis of variance (ANOVA) (Table 2). P-values < 0.05 showed that the model is statistically significant [4]. Five variables (initial AMX concentration, pyrite dose, per sulfate concentration, time and pH) had linearly significant effect with p-value < 0.05. The R- Squared value (0.9828) is close to adjusted R-squared (0.9877) implying high importance of the model [5]. The diagrams of normal probability of the studentized residuals and the predicted against experimental values are shown in Fig. 1, Fig. 2, respectively. Fig. 3 shows the interaction effects of variables on AMX removal efficiency. According to the results, a quadratic equation between dependent variable (AMX removal %) and independent variables was obtained as follows:

**Table 1:** Levels of independent variables and experimental range in Box-Behnken design.

Factors	Range and level		
	−1	0	+1
A: Initial AMX (mg/l)	10	40	70
B: catalyst load (g/L)	1	2	3
C: per sulfate concentration (mM)	0.5	2	3.5
D: Time(min)	30	45	60
E:pH	3	6	9

**Table 2:** ANOVA test for quadratic model.

Source	Sum of squares	Degree of freedom	Mean square	*F* value	*P*-value	
					Prob>*F*	
Model	14,539.55	13	111.43	198.39	< 0.0001	Significant
A	1440.1	1	1440.1	255.44	< 0.0001	Significant
B	41.93	1	41.93	7.44	0.0103	Significant
C	603.56	1	603.56	107.6	< 0.0001	Significant
D	7428.72	1	7428.72	1317.74	< 0.0001	Significant
E	3603	1	3603	639.12	< 0.0001	Significant
AD	21.58	1	21.58	3.83	0.0479	Significant
BD	22.52	1	22.52	3.99	0.0490	Significant
CD	0.22	1	0.22	0.038	0.0592	Not Significant
A^2^	49.09	1	49.09	8.71	< 0.0059	Significant
B^2^	890.71	1	890.71	158	< 0.0001	Significant
C^2^	168.96	1	168.96	29.97	< 0.0268	Significant
D^2^	30.48	1	30.48	5.93	< 0.0241	Significant
E^2^	31.59	1	31.59	5.60	< 0.0278	Significant
Residual	180.40	32	5.64			
Lack of Fit	142.54	27	5.28	0.70	0.7559	Not significant
Pure Error	37.86	5	7.57			
Cor Total	14,719.95	45				
R-square	0.9877					
Adj R-square	0.9828					
Pred R-squared	0.9700					
Adequate precision	55.813

$AMX\;removal\left(\%\right)=55.35–9.49A–1.62B+6.14C+21.55D+15.01E+2.32AD–2.37BD–0.23CD+2.37A^{2}–10.10B^{2}–4.40C^{2}+1.87D^{2}–1.90E^{2}$

## 2 Experimental design, materials and methods

### 2.1 Materials

AMX (CAS 26787-78-0) and Sodium persulfate (Na_2_S_2_O_8_ 98%) were obtained from Sigma- Aldrich. FeS_2_ rock sample (Pyrite) was purchased from Department of Mine Engineering, university of Tehran.

### 2.2 Catalyst preparation

Firstly, pyrite rock sample by a ceramic mortar was milled and for 5 min in ethanol (95%) was ultra-sonicated. For removal of impurities was washed with 1 M nitric acid, rinsed with deionized water and ethanol, respectively. Subsequently, pyrite was dried at 30 °C. Finally, pyrite was sieved (80 µm) [6].

### 2.3 Determination of AMX concentration

The AMX concentration of all samples was measured by A High Liquid Performance Chromatography (HPLC, CE4200-cecil, England). The equation below was applied for obtaining the removal efficiency (ƞ %) as follows [7], [8], [9]:

(2) $$\left(\frac{C_{0}-C_{F}}{C_{0}}\right)\times 100\%$$

Where, C_0_ is the initial concentration and C_t_ is residual concentration of AMX.

### 2.4 Experimental design

#### 2.4.1 Box-Behnken design experiments

The experiments designed by Design-Expert software (version 7), based on Box*–*Behnken design (BBD) and total experiments were 46 runs. BOX-Behnken design was used to analyze five parameters i.e. pH, concentration of per sulfate, Fe S_2_ concentration, contact time and initial AMX concentration on AMX removal efficiency and removal optimum conditions.

#### 2.4.2 AMX removal experiments

Firstly, the stock solution of 1000 mg/L AMX was prepared to obtain different concentration. Then,the effects of variables such as initial AMX (10–80 mg/L), solution pH (3–9), contact time (30–60 min), pyrite dose (1–3 g/L) and persulfate concentration (0.5–3.5 mM) were evaluated.

## Funding sources

This research was supported by Iran University of Medical Sciences under Grant no. 30049.
